# Digital Smoking Cessation Intervention for Cancer Survivors: Analysis of Predictors and Moderators of Engagement and Outcome Alongside a Randomized Controlled Trial

**DOI:** 10.2196/46303

**Published:** 2024-06-20

**Authors:** Rosa Andree, Ajla Mujcic, Wouter den Hollander, Margriet van Laar, Brigitte Boon, Rutger Engels, Matthijs Blankers

**Affiliations:** 1 Trimbos Institute Netherlands Institute of Mental Health and Addiction Utrecht Netherlands; 2 PsyQ Parnassia Groep The Hague Netherlands; 3 Siza Center for Long-term Care for People with Disabilities Arnhem Netherlands; 4 Academy Het Dorp Research & Advisory on Technology in Long-term Care Arnhem Netherlands; 5 Tranzo Tilburg School of Social and Behavioral Sciences Tilburg University Tilburg Netherlands; 6 Erasmus School of Social and Behavioural Sciences Erasmus University Rotterdam Rotterdam Netherlands; 7 Department of Research Arkin Mental Health Care Amsterdam Netherlands

**Keywords:** smoking cessation, cancer survivors, engagement, digital intervention, eHealth, smoking, intervention, randomized controlled trial, predictor, RCT, smoking, smoker, addict, cessation, quit, cancer, oncology

## Abstract

**Background:**

Recent studies have shown positive, though small, clinical effects of digital smoking cessation (SC) interventions for cancer survivors. However, research on associations among participant characteristics, intervention engagement, and outcomes is limited.

**Objective:**

This study aimed to explore the predictors and moderators of engagement and outcome of MyCourse-Quit Smoking (in Dutch: “MijnKoers-Stoppen met Roken”), a digital minimally guided intervention for cancer survivors.

**Methods:**

A secondary analysis of data from the randomized controlled trial was performed. The number of cigarettes smoked in the past 7 days at 6-month follow-up was the primary outcome measure. We analyzed interactions among participant characteristics (11 variables), intervention engagement (3 variables), and outcome using robust linear (mixed) modeling.

**Results:**

In total, 165 participants were included in this study. Female participants accessed the intervention less often than male participants (B=–11.12; *P*=.004). A higher Alcohol Use Disorders Identification Test score at baseline was associated with a significantly higher number of logins (B=1.10; *P*<.001) and diary registrations (B=1.29; *P*<.001). A higher Fagerström Test for Nicotine Dependence score at baseline in the intervention group was associated with a significantly larger reduction in tobacco use after 6 months (B=–9.86; *P*=.002). No other associations and no moderating effects were found.

**Conclusions:**

Overall, a limited number of associations was found between participant characteristics, engagement, and outcome, except for gender, problematic alcohol use, and nicotine dependence. Future studies are needed to shed light on how this knowledge can be used to improve the effects of digital SC programs for cancer survivors.

**Trial Registration:**

Netherlands Trial register NTR6011/NL5434; https://onderzoekmetmensen.nl/nl/trial/22832

## Introduction

### Background

In the past decade, digital interventions have commonly been used to target addictive behaviors, including smoking cessation (SC). Several systematic reviews have shown that these SC interventions can be effective, albeit with generally small effect sizes [1-4]. For example, the Cochrane review by Taylor et al [4] showed that the use of web-based SC interventions resulted in significantly higher rates of smoking abstinence compared to nonactive control groups, 6 months after randomization (risk ratio=1.15). Cancer survivors are a growing population who can benefit considerably from SC. Yet, the prevalence of people who smoke is about the same as in the general population, and research on effective digital SC interventions for cancer survivors is scarce [3]. Accordingly, not much is known about active ingredients or engagement factors of SC interventions targeting cancer survivors [5], despite engagement being an important moderator of the effect of digital SC interventions [6]. It is therefore important to look more closely into the predictors and moderators of engagement and outcome among this target group.

Although the primary effects of digital SC interventions are moderately positive on average, there is room for improvement. One possible explanation for the modest effects of digital SC interventions is the generally low adherence rates. Taylor et al [4] found that 18 out of 34 web-based SC studies had more than 50% attrition at follow-up. Analyzing whether the uptake of specific intervention components is related to better intervention outcomes increases the understanding of primary intervention effects [7]. Some studies on addictive behaviors investigated the relationship between intervention engagement and outcome [8-10]. A study by Perski et al [8] found that participants who completed more (varied) exercises had 64% higher odds of SC compared to participants who almost exclusively set an SC goal. Siemer et al [10], examining adherence to a blended SC intervention, revealed a dose-response relationship between the number of executed activities and smoking abstinence. Another study by Ramos et al [9] also found that intervention engagement, in terms of number of logins, forum visits, and number of participation badges, was a strong predictor of successful SC. Not all studies have shown that intervention engagement predicts intervention effectiveness, even contradictory effects are found. For example, Smith et al [11] showed that engagement with particular components of a digital SC intervention can be counterproductive when the content does not fit the participants’ needs.

Behavior change programs targeting SC notoriously encounter challenges when trying to reach target groups with the highest smoking rates (eg, groups with lower socioeconomic status [12] and groups with low literacy [13]). In addition, it could be useful for the improvement of intervention content and implementation to identify which characteristics predict engagement. This will help to improve the content and design of the intervention for the right target group [7,14]. Several studies have related digital SC intervention use to participant characteristics [8,15-17]. These studies showed that digital SC interventions were used longer or more frequently by older participants [8,15] and women [16]. Participants who had lower education, smoked more heavily, and had depressive symptoms were found to be less engaged with the digital SC intervention [17].

There is some evidence on the effects of digital SC interventions for cancer survivors. For example, a meta-analytic study by Mujcic et al [3] showed that digital and nondigital distance-based SC interventions for cancer survivors led to significantly reduced smoking rates compared to baseline (risk difference=0.29). However, research on the predictors and moderators of engagement and outcome of digital SC studies for cancer survivors is limited, while cancer survivors are a growing and diverse population [18]. A pilot study by Bricker et al [18] of an application on SC for cancer survivors showed greater acceptability, use, and effectiveness when compared to the national SC app for the general population.

### This Study

In this study, we aim to investigate the predictors and moderators of engagement and outcome of a minimally guided digital intervention for cancer survivors called MyCourse-Quit Smoking (in Dutch “MijnKoers-Stoppen met Roken”) in a secondary analysis. The main effects study did not find a differential effect on SC between intervention and control at 6 months. In both groups, around a quarter abstained from smoking, and the number of cigarettes smoked was cut back by half [19]. With this secondary analysis, we aim to answer the following research questions: (1) Are participant characteristics related to intervention engagement at 6-month follow-up? (2) Is intervention engagement associated with tobacco use at 6-month follow-up? (3) Are participant characteristics related to tobacco use at 6-month follow-up?

## Methods

### Design

For this paper, an exploratory secondary data analysis was carried out using data from a randomized controlled trial on the MyCourse-Quit Smoking digital intervention. The data used for this study were collected between November 2016 and September 2019.

### Ethical Considerations

Ethics approval for the trial was acquired from an accredited medical research and ethics committee in The Netherlands (Toetsingscommissie Wetenschappelijk Onderzoek Rotterdam e.o. NL55921.101.16). Participants provided digital informed consent before inclusion in the trial [20]. Data were deidentified before processing or analysis. Identifying data were stored separately from research data. For each completed follow-up assessment, they were reimbursed €25 (approximately US $30).

### Procedure

A web-based screening questionnaire on the study website determined whether people were able to participate in the study. Eligible participants received an informed consent form via mail and had 30 days to sign the form. In the meantime, participants had the possibility to contact the research team or an independent physician for more information. After signing the informed consent form, they were asked to fill out a baseline questionnaire and were allocated to either the MyCourse group or the control group. Individuals in the control group were provided access to a noninteractive web-based informational brochure regarding the hazards associated with smoking and strategies for SC. The informational content encompassed both general SC information and content tailored to the unique needs of cancer survivors. Follow-up measurements were conducted at 3, 6, and 12 months after randomization. The study was conducted completely over the web, but after continued nonresponse, participants received a reminder by telephone. A more extensive description of the randomized controlled trial study procedures can be found elsewhere [20].

### Participants

For the study, 165 adults who were diagnosed with any form of cancer in the past 10 years were recruited. Other eligibility criteria included having a PC or laptop in addition to an internet connection at home, having smoked 5 or more cigarettes per day in the past 7 days, having the intention and ability to participate in the 12-month study, and having the intention to quit smoking cigarettes. People who were pregnant; had insufficient mastery of the Dutch language; or self-reported suicidal ideation, dementia, severe depression, severe alcohol dependence, or acute psychosis were not eligible to participate in the study.

### MyCourse Intervention

MyCourse-Quit Smoking is a digital minimally guided intervention that provides support for SC among cancer survivors. The intervention is based on empirically evaluated therapeutic approaches for SC in the general population: cognitive behavioral therapy [21], motivational interviewing [22], and acceptance and commitment therapy [23]. The intervention can be accessed via PC, tablet, and smartphone. At first login, participants receive instructions to set up a quit plan and gain access to 13 different exercises, information about smoking, quitting, and cancer, a web-based diary to track their tobacco use, and a peer support platform [20]. Exercises focused on different topics including previous experiences, high-risk situations, self-control measures, reinforcement, relapse prevention, and acceptance and commitment therapy. For the complete structure of the intervention, see Figure 3 in the protocol paper [20]. After the first login, all parts of the intervention could be accessed, and participants were free to choose how often and which parts of the intervention they wanted to use. Participants were only advised to use the intervention daily for 4 weeks.

### Measures

The primary outcome measure in this study was the number of cigarettes smoked in the past 7 days at 6-month follow-up. Intervention engagement was measured using 3 indicators: the number of logins into the MyCourse-Quit Smoking intervention, the number of self-monitoring registrations of smoking urges and smoked cigarettes, and the number of completed intervention exercises. The following participant characteristics were extracted from the participant records: gender, age, educational level (higher or lower, where the minimum for the higher educational level was an academic university or university of applied sciences degree), and living situation (alone or together). We specifically looked at the presence of lung cancer and breast cancer (yes or no) among the participants in the analyses because lung cancer has a direct relationship with smoking and breast cancer was the most common type of cancer in the sample. In addition, patients with cancer at other sites were included in the analyses. Furthermore, the number of cancer sites (1 or >1) distinguished participants who reported that they had received multiple cancer diagnoses. The severity of nicotine dependence was measured by the 6-item Fagerström Test for Nicotine Dependence (FTND) [24]. Problematic alcohol use was measured using the Alcohol Use Disorders Identification Test (AUDIT) [25], a 10-item questionnaire on alcohol consumption patterns and problems experienced due to alcohol consumption. The AUDIT score was included as a variable because research has shown that people with a high risk of problematic alcohol use have a harder time quitting smoking and may benefit from different types of SC treatment [26,27]. The EQ-5D was used to measure the quality of life [28]. Comorbid anxiety, depression, and somatic symptoms were indicated using the Brief Symptom Inventory-18 questionnaire [29].

### Statistical Analysis

#### Imputation of Missing Data

Missing data for primary (ie, cigarettes smoked in the past 7 days) and secondary (ie, participant characteristics) outcome measures were multiple imputed (number of imputations=50) based on the intention-to-treat principle using the predictive mean matching method from the mice package in R (R Foundation for Statistical Computing) [29]. At the 6-month follow-up, the nonresponse rate (ie, participants who did not complete the 6-month questionnaire) was 27.7% (23/83) in the intervention group and 25.6% (21/82) in the control group. Data on intervention usage were not imputed. For the analyses containing engagement measures, participants who did not log in once were excluded.

#### Regression Analyses

Data were analyzed using R [30]. Bonferroni correction was applied in all analyses. The association between intervention engagement and participant characteristics within the intervention group was analyzed with a robust linear regression using the MASS package [31]. Whether participant characteristics and intervention engagement predicted intervention outcome within the intervention group was analyzed using robust linear mixed modeling (RLMM) with a random intercept using the robustlmm package [32]. RLMM is an effective analytical approach to account for outliers or skewed data [32]. The moderation analyses to investigate whether the study condition moderated the effect between participant characteristics and outcome were performed using RLMM with a random intercept and study condition × participant characteristics as the interaction term. This analysis is performed to assess whether the study condition (ie, being in the intervention group compared to the control group) moderates the association between participant characteristics and outcome. Model estimates, 95% CIs, and *P* values are reported. All base case analyses followed the intention-to-treat principle and used multiple imputed data sets. Sensitivity analyses were performed using observed data only.

## Results

### Sample Characteristics

Participant characteristics are shown in Table 1. The majority of participants were female (136/165, 82.4%), the mean age was 54.2 (SD 11.2) years, 29.1% (48/165) were living alone, and 41.2% (68/165) had completed higher education. On average, participants had smoked for 34.5 (SD 12.0) years and smoked 100 (SD 51.2) cigarettes per week. The main clinical effects of the MyCourse intervention and the results of the cost-effectiveness analysis can be found elsewhere [19].

**Table 1:** Baseline characteristics.

Characteristics		MyCourse (n=83)^a^	Control (n=82)	Total (N=165)^a^
**Sex, n (%)**				
	Female	70 (84.3)	66 (80.5)	136 (82.4)
	Male	13 (15.7)	16 (19.5)	29 (17.6)
Age (years), mean (SD)		55.0 (12.1)	53.3 (10.3)	54.2 (11.2)
**Higher education, n (%)**				
	Yes	25 (30.1)	19 (23.2)	44 (26.7)
	No	49 (59.0)	48 (58.5)	97 (58.8)
**Living situation, n (%)**				
	Living alone	22 (26.5)	26 (31.7)	48 (29.1)
	Living together	61 (73.5)	56 (68.3)	117 (70.9)
**Smoking behavior** **, mean (SD)**				
	Years smoked	34.4 (11.8)	34.6 (12.2)	34.5 (12)
	Number of cigarettes in past 7 days	101.8 (54.3)	98.2 (48.2)	100 (51.2)
	FTND^b^	4.9 (2.4)	4.9 (2.3)	4.9 (2.4)
**Drinking behavior**				
	Drank alcohol in last month, n (%)	55 (66.3)	55 (67.1)	110 (66.7)
	Number of drinks in past 7 days, mean (SD)	6.9 (13.1)	5.6 (8.7)	6.2 (11.2)
	AUDIT^c^, mean score (SD)	3.7 (5.1)	3.6 (4.2)	3.6 (4.7)
**Cancer diagnosis,** ^d^ **n (%)**				
	Breast	42 (42.9)	33 (34)	75 (38.5)
	Lung	14 (14.3)	9 (9.3)	23 (11.8)
	Uterine	7 (7.1)	12 (12.4)	19 (9.7)
	Head and neck	10 (10.2)	8 (8.2)	18 (9.2)
	Colon	5 (5.1)	5 (5.2)	10 (5.1)
	Other (including bladder, lymphatic, melanoma, skin, kidney, prostate)	20 (20.4)	30 (30.9)	50 (25.6)
**Cancer sites, n (%)**				
	1	69 (83.1)	68 (82.9)	137 (83)
	2 or 3	14 (16.9)	14 (17.1)	28 (17)

### Participant Characteristics and Intervention Engagement

Of all 83 participants of the intervention group, 56 people logged into the MyCourse portal at least once and thus were included in the analysis. When comparing the 56 people who logged in at least once with the 27 people who did not log in once at all baseline characteristics mentioned in Table 1, only the number of patients with uterine cancer differed significantly between the 2 groups (*P*<.05), with 5 patients with uterine cancer who did not log in once and 2 patients with uterine cancer that logged in at least once. In total, 82 participants in the control group were not included in the analysis. Among the 56 MyCourse users, the average number of logins was 21 (SD 41.0; median 5.5, IQR 3-18.5), the average amount of self-monitoring registrations was 31 (SD 53.9; median 5, IQR 2-22), and the average amount of completed exercises was 6.5 (SD 5.1; median 4, IQR 2-12). As shown in Table 2, female participants showed a significantly lower number of logins in the MyCourse-Quit Smoking intervention than male participants (*P*=.004). The relationship between sex and other indicators of intervention engagement was nonsignificant. Furthermore, a higher AUDIT score at baseline was associated with a significantly higher number of logins (*P*<.001) and diary registrations (*P*<.001) but not with the number of completed exercises (*P*=.05).

**Table 2:** The association between baseline participant characteristics and intervention engagement (N=56).

		Logins		Diary entries		Exercises	
		B (95% CI)	*P* values^a^	B (95% CI)	*P* values ^a^	B (95% CI)	*P* values ^a^
Age (years)		0.25 (–0.10 to 0.60)	.16	0.40 (–0.16 to 0.96)	.16	0.08 (–0.06 to 0.22)	.28
**Sex**							
	Male (n=8)	Reference	Reference	Reference	Reference	Reference	Reference
	Female (n=48)	–11.12 (–18.70 to –3.55)	*.004* ^b^	–11.85 (–24.25 to 0.54)	.06	–2.24 (–6.43 to 1.94)	.29
**Higher education**							
	No (n=40)	Reference	Reference	Reference	Reference	Reference	Reference
	Yes (n=16)	3.08 (–4.10 to 10.26)	.40	4.43 (–4.71 to 13.57)	.34	1.71 (–1.24 to 4.67)	.26
**Living situation**							
	Alone (n=11)	Reference	Reference	Reference	Reference	Reference	Reference
	Together (n=45)	0.32 (–7.46 to 8.11)	.94	0.72 (–10.64 to 12.08)	.90	–0.43 (–3.90 to 3.04)	.81
FTND^c^		–0.15 (–1.51 to 1.22)	.83	–0.33 (–2.32 to 1.66)	.75	0.15 (–0.46 to 0.75)	.64
EQ-5D		–4.88 (–21.59 to 11.84)	.57	–9.43 (–33.30 to 14.44)	.44	–4.25 (–11.37 to 2.87)	.24
BSI-18^d^		–3.57 (–8.36 to 1.23)	.15	–5.07 (–11.46 to 1.32)	.12	–1.30 (–3.48 to 0.87)	.24
AUDIT^e^		1.10 (0.60 to 1.61)	*<.001* ^f^	1.29 (0.62 to 1.95)	*<.001*	0.25 (0.00 to 0.50)	.05
**Diagnosis of lung cancer**							
	No (n=47)	Reference	Reference	Reference	Reference	Reference	Reference
	Yes (n=9)	–2.19 (–10.48 to 6.09)	.60	–3.01 (–15.04 to 9.02)	.62	1.30 (–2.36 to 4.96)	.49
**Diagnosis of breast cancer**							
	No (n=25)	Reference	Reference	Reference	Reference	Reference	Reference
	Yes (n=31)	3.70 (–2.59 to 9.99)	.25	5.94 (–1.87 to 13.74)	.14	1.65 (–1.07 to 4.37)	.24
**Cancer sites**							
	1 (n=47)	Reference	Reference	Reference	Reference	Reference	Reference
	2 or 3 (n=9)	–2.75 (–11.29 to 5.79)	.53	–3.88 (–17.73 to 9.98)	.58	0.64 (–3.04 to 4.31)	.73

^a^A Bonferroni correction was applied based on 11 tests resulting in an α of .0045.

^b^Female participants showed a significantly lower number of logins in the MyCourse-Quit Smoking intervention than male participants.

^c^FTND: Fagerström Test for Nicotine Dependence.

^d^BSI-18: Brief Symptom Inventory-18.

^e^AUDIT: Alcohol Use Disorders Identification Test.

^f^A higher AUDIT score at baseline was associated with a significantly higher number of logins and diary registrations but not with the number of completed exercises.

### Intervention Engagement, Participant Characteristics, and Smoking Behavior

Table 3 shows the outcomes of the analysis on the association between intervention engagement and smoking behavior among the 56 participants who logged in to the MyCourse portal at least once. No significant effects were found between intervention engagement and smoking behavior. Table 3 also shows the association between several participant characteristics and smoking behavior among the 83 participants of the intervention group. The results show that a higher FTND score at baseline is associated with a significantly greater reduction of the 7-day sum of smoked cigarettes after 6 months in the intervention group (*P*=.002). None of the other participant characteristics or measures of engagement predicted smoking behavior at 6 months.

**Table 3:** The relationship between participant characteristics and intervention engagement with smoking behavior.

Characteristics			Effect on 7-day tobacco use at 6-month follow-up			
			B (95% CI)		*P* values^a^	
Age (years) (n=83)			0.70 (–0.89 to 2.29)		.39	
**Sex**						
	Male (n=13)	Reference		Reference		
	Female (n=70)	–5.71 (–51.07 to 39.65)		.81		
**Higher education**						
	No (n=58)	Reference		Reference		
	Yes (n=25)	–2.16 (–37.12 to 32.80)		.90		
**Living situation**						
	Alone (n=22)	Reference		Reference		
	Together (n=61)	–1.04 (–38.13 to 36.05)		.96		
FTND^b^ (n=83)			–9.86 (–15.95 to –3.76)		*.002* ^c^	
EQ-5D (n=83)			17.28 (–64.19 to 98.74)		.68	
BSI-18^d^ (n=83)			1.25 (–27.43 to 29.94)		.93	
AUDIT^e^ (n=83)			2.38 (–0.76 to 5.53)		.14	
**Diagnosis of lung cancer**						
	No (n=69)	Reference		Reference		
	Yes (n=14)	19.84 (–10.95 to 50.62)		.21		
**Diagnosis of breast cancer**						
	No (n=41)	Reference		Reference		
	Yes (n=42)	11.77 (–32.27 to 55.81)		.60		
**Cancer sites**						
	1 (n=69)	Reference		Reference		
	2 or 3 (n=14)	53.77 (13.70 to 93.83)		.009		
Number of logins (n=56)			–0.13 (–0.55 to 0.30)		.56	
Number of diary entries (n=56)			–0.02 (–0.35 to 0.30)		.88	
Number of exercises (n=56)			0.19 (–3.31 to 3.69)		.92	

^a^A Bonferroni correction was applied based on 14 tests resulting in an α of .004.

^b^FTND: Fagerström Test for Nicotine Dependence.

^c^A higher FTND score at baseline is associated with a significantly greater reduction of the 7-day sum of smoked cigarettes after 6 months in the intervention group.

^d^BSI-18: Brief Symptom Inventory-18.

^e^AUDIT: Alcohol Use Disorders Identification Test.

### Moderation Analysis

Table 4 reports the outcomes of the moderation analysis on the interaction effect of participant characteristics and study condition on the number of cigarettes smoked in the past 7 days among the 165 participants at 6-month follow-up. No significant effects were found in this analysis.

**Table 4:** Moderation analysis of study condition on the relationship between participant characteristics and smoking behavior.

Characteristic		Participant characteristic × randomized controlled trial condition 7-day tobacco use at 6-month follow-up (N=165)	
		B (95% CI)	*P* values^a^
Age (years)		1.229 (–1.06 to 3.52)	.29
**Sex**			
	Male (n=29)	Reference	Reference
	Female (n=136)	–11.7 (–73.76 to 50.36)	.71
**Higher education**			
	No (n=121)	Reference	Reference
	Yes (n=44)	–25.21 (–77.18 to 26.76)	.34
**Living situation**			
	Alone (n=48)	Reference	Reference
	Together (n=117)	7.989 (–43.59 to 59.57)	.76
FTND^b^		–3.624 (–12.20 to 4.95)	.41
EQ-5D		45.117 (–75.26 to 165.49)	.46
BSI-18^c^		–12.024 (–55.66 to 31.61)	.59
AUDIT^d^		4.155 (–1.04 to 9.35)	.12
**Diagnosis of lung cancer**			
	No (n=142)	Reference	Reference
	Yes (n=23)	42.98 (–24.59 to 110.55)	.21
**Diagnosis of breast cancer**			
	No (n=90)	Reference	Reference
	Yes (n=75)	9.25 (–36.89 to 55.39)	.69
**Cancer sites**			
	1 (n=137)	Reference	Reference
	2 or 3 (n=28)	81.71 (22.50 to 140.91)	.007

^a^A Bonferroni correction was applied based on 11 tests resulting in an α of .0045.

^b^FTND: Fagerström Test for Nicotine Dependence.

^c^BSI-18: Brief Symptom Inventory-18.

^d^AUDIT: Alcohol Use Disorders Identification Test.

### Sensitivity Analysis

The association between sex and the number of logins on the nonimputed data did not reach significance after the Bonferroni correction (*P*=.006). The association between the FTND and 7-day cigarette smoking at 6-month follow-up in the nonimputed data did not reach significance after Bonferroni correction (*P*=.10). In the moderation analysis, after Bonferroni correction on the nonimputed data, the interaction effect of the number of cancer sites and study condition on smoking behavior reached significance (*P*=.002). For all other analyses, the results did not change significantly. Detailed results of the sensitivity analyses can be found in Multimedia Appendix 1.

## Discussion

### Principal Findings

In this study, we evaluated hypothesized predictors and moderators of intervention engagement and smoking behavior in MyCourse-Quit Smoking, a digital SC intervention for cancer survivors. With regard to the relationship between participant characteristics and intervention engagement, it was found that female participants logged on significantly less often than male participants. This effect should nevertheless be interpreted with caution since the number of male participants in the sample was low (n=8). Moreover, previous research shows that female participants are generally more engaged in digital SC interventions than male participants [16,33-37]. A significant positive association between the baseline AUDIT score and intervention engagement was found; a higher AUDIT score at baseline was related to a higher number of logins and diary registrations in the MyCourse intervention. There was no effect of the baseline AUDIT score on the number of completed exercises. Previous studies showed that participants with a higher risk of alcohol dependence had a harder time to quit smoking, and therefore needed more support from the intervention, as demonstrated in several previous studies [26,27]. Toll et al [27] showed that people who drink more heavily were less likely to quit smoking, but problematic alcohol use was not measured. Sells et al [26] pointed out that people with a high risk of problematic alcohol use may need more intensive intervention in order to quit smoking, whereas people with a high risk of problematic alcohol use were defined with an AUDIT score higher than 7. However, in this study, we did not find an effect of the AUDIT score on smoking behavior. Furthermore, participants of the MyCourse-Quit Smoking trial had generally low AUDIT scores (mean 3.6, SD 4.7), and few participants with a score higher than 7 (21/165).

Regarding the association between participant characteristics and smoking behavior, we found that participants of the MyCourse intervention who had higher nicotine dependence scores at baseline showed a greater reduction in the number of smoked cigarettes in the past 7 days at the 6-month follow-up. This negative association between nicotine dependence at baseline and tobacco use at follow-up is a reasonable finding because it is likely that less addicted participants at baseline already smoke fewer cigarettes than highly addicted participants, and therefore, a smaller reduction of cigarettes at 6 months is possible. This finding does not indicate whether heavier nicotine dependence predicts SC, as participants can greatly reduce the number of smoked cigarettes but not enough to completely quit smoking. Previous research shows that, in general, less severe nicotine dependence is associated with a higher SC rate [38,39].

The analyses on the association between intervention engagement (ie, the number of logins, self-monitoring registrations, and exercises) and the outcome did not yield any significant effects. This study showed the overall prevailing pattern of the majority of participants quitting the use of the intervention in the first few days and a smaller group that uses the intervention for a longer period [40]. However, other studies on digital SC interventions have shown a dose-response relationship between intervention engagement and outcome [9,10,41], with higher engagement predicting greater SC rates, although this is sometimes limited to certain engagement measures [34] or with low quality of the evidence due to low follow-up rates [9]. For example, Heminger et al [34] did not find a significant association between program dose and SC, but the use of specific intervention elements (eg, making a pledge toward a smoke-free life and tracking saved money and health benefits gained after quitting) was associated with SC. For future research, it is therefore important to properly define engagement, differentiate between indicators of engagement, and use empirically effective intervention techniques in order to enhance engagement [6].

The moderation analysis did not yield any significant effects. This indicates that being in the intervention group, compared to the control group, does not amplify the effect between any of the participant’s characteristics and tobacco use, and hence no specific participant characteristic renders participants more or less likely to be successful when participating in the MyCourse intervention.

### Limitations

The initial study was 80% powered to detect a relative risk of 2.1 in SC [20], while this explored different outcome variables, potential moderator effects herein, and made comparisons other than between treatment arms. Hence, the initial sample size calculation might not be applicable. Post hoc power analyses were omitted, as these would merely reflect the already obtained *P* value [42]. While the applied Bonferroni correction accounted for multiple comparisons, it might be overly strict in our case [43]. Furthermore, the tendency to overfit data might also be a problem for linear mixed modeling analyses. The study had missing data, which might have caused bias in the results. On the other hand, as a strength of this study, multiple imputation was applied to compensate for the missing values, and the sensitivity analysis did not reveal any substantial differences in the analyses without imputation. Another limitation is the sample size of the analyses for the first research question, especially for the subgroup analyses of sex and living situation. Since some of the categories of these variables had small group sizes, the outcomes of the analyses should be interpreted with caution.

### Clinical Implications

The MyCourse intervention is presumably more engaging for people who smoke and people with moderate to high alcohol dependence. Furthermore, this study did not identify any specific subgroups where the MyCourse-Quit Smoking intervention might be particularly effective or ineffective.

### Conclusions

This study aimed to provide more insight into predictors and moderators of engagement and outcome for a digital SC intervention targeting cancer survivors. Overall, a limited number of associations was found between participant characteristics, engagement, and smoking behavior. Female participants accessed the intervention less often than male participants, and participants with higher AUDIT scores accessed the intervention more often and had more diary registrations than participants with lower AUDIT scores. Greater nicotine dependence at baseline was associated with a greater reduction in number of cigarettes at 6 months. Future studies in a larger sample and with a preregistered analysis plan are needed to corroborate these findings and shed light on how this knowledge can be used to improve the effects of digital SC programs.

## Appendix

Multimedia Appendix 1 Sensitivity analysis of all performed analyses.

## Abbreviations

AUDIT: Alcohol Use Disorders Identification Test
FTND: Fagerström Test for Nicotine Dependence
RLMM: robust linear mixed modeling
SC: smoking cessation
