# Supplementary material for: Digital Smoking Cessation Intervention for Cancer Survivors: Analysis of Predictors and Moderators of Engagement and Outcome Alongside a Randomized Controlled Trial
Source: JMIR Cancer. 2024 Jun 20;10:e46303. doi: 10.2196/46303 (PMC11229662; doi:10.2196/46303)
Supplement: Multimedia Appendix 1 [file cancer_v10i1e46303_app1.docx]

Table 1. Sensitivity analysis of the association between baseline participant characteristics and intervention engagement.

|  |  |  | Number of logins (N = 56) | | | Number of diary entries (N = 56) | | | Number of exercises (N = 56) | | |
| --- | --- | --- | --- | --- | --- | --- | --- | --- | --- | --- | --- |
|  |  | n | B | CI | *P*^a^ | B | CI | *P*^a^ | B | CI | *P*^a^ |
| **Age (years)** | |  |  |  |  |  |  |  |  |  |  |
|  | |  | 0.05 | -0.20, 0.29 | .697 | 0.12 | -0.24, 0.47 | .508 | 0.02 | -0.09, 0.13 | .715 |
| **Gender** | |  |  |  |  |  |  |  |  |  |  |
|  | Men | 8 | REF^b^ | REF | REF | REF | REF | REF | REF | REF | REF |
|  | Women | 48 | -11.12 | -18.87, -3.37 | .006 | -11.85 | -24.53, 0.83 | .066 | -2.24 | -6.52, 2.04 | .298 |
| **Higher education** | |  |  |  |  |  |  |  |  |  |  |
|  | No | 40 | REF | REF | REF | REF | REF | REF | REF | REF | REF |
|  | Yes | 16 | 3.08 | -4.27, 10.43 | .404 | 4.43 | -4.92, 13.78 | .346 | 1.71 | -1.31, 4.73 | .261 |
| **Living situation** | |  |  |  |  |  |  |  |  |  |  |
|  | Alone | 11 | REF | REF | REF | REF | REF | REF | REF | REF | REF |
|  | Together | 45 | -0.42 | -8.92, 8.09 | .922 | 0.09 | -13.28, 13.45 | .989 | -0.67 | -4.34, 3.00 | .717 |
| **FTND** | |  |  |  |  |  |  |  |  |  |  |
|  | |  | -0.15 | -1.54, 1.25 | .832 | -0.33 | -2.36, 1.71 | .747 | 0.15 | -0.47, 0.76 | .637 |
| **EQ5D** | |  |  |  |  |  |  |  |  |  |  |
|  | |  | -4.31 | -21.96, 13.34 | .626 | -8.90 | -34.21 – 16.42 | .484 | -3.93 | -11.12, 3.26 | .278 |
| **BSI-18** | |  |  |  |  |  |  |  |  |  |  |
|  | |  | -3.56 | -8.47, 1.34 | .151 | -5.07 | -11.60-1.46 | .126 | -1.30 | -3.53, 0.92 | .245 |
| **AUDIT** | |  |  |  |  |  |  |  |  |  |  |
|  | |  | 1.10 | 0.59, 1.62 | *<.001* | 1.29 | 0.61-1.97 | *<.001* | 0.25 | 0.00, 0.50 | .054 |
| **Diagnosis lung cancer** | |  |  |  |  |  |  |  |  |  |  |
|  | No | 47 | REF | REF | REF | REF | REF | REF | REF | REF | REF |
|  | Yes | 9 | -2.19 | -10.67, 6.28 | .606 | -3.01 | -15.31, 9.29 | .626 | 1.30 | -2.44, 5.05 | .489 |
| **Diagnosis breast cancer** | |  |  |  |  |  |  |  |  |  |  |
|  | No | 25 | REF | REF | REF | REF | REF | REF | REF | REF | REF |
|  | Yes | 31 | 3.70 | -2.73, 10.14 | .254 | 5.93 | -2.05, 13.92 | .142 | 1.65 | -1.14, 4.44 | .241 |
| **Cancer sites** | |  |  |  |  |  |  |  |  |  |  |
|  | 1 | 47 | REF | REF | REF | REF | REF | REF | REF | REF | REF |
|  | 2 or 3 | 9 | -2.75 | -11.48, 5.99 | .531 | -3.87 | -18.05, 10.30 | .586 | 0.64 | -3.12, 4.40 | .735 |

^a^A Bonferroni correction was applied based on 11 tests resulting in an alpha of 0.0045.
^b^REF: reference category.

Table 2. Sensitivity analysis of the relationship between participant characteristics and intervention engagement with smoking behavior.

|  | |  |  | |  | Effect on 7-day tobacco use at 6-month follow-up | | |  |
| --- | --- | --- | --- | --- | --- | --- | --- | --- | --- |
|  |  | | | n | | B | CI | *P*^a^ |  |
| **Age (years)** | | | |  | |  |  |  |  |
|  |  | | | 83 | | 0.92 | -0.66, 2.53 | .262 |  |
| **Gender** | | | |  | |  |  |  |  |
|  | Men | | | 13 | | REF^b^ | REF | REF |  |
|  | Women | | | 70 | | 30.01 | -21.17, 82.22 | .261 |  |
| **Higher education** | | | |  | |  |  |  |  |
|  | No | | | 58 | | REF | REF | REF |  |
|  | Yes | | | 25 | | 18.39 | -18.02, 55.70 | .332 |  |
| **Living situation** | | | |  | |  |  |  |  |
|  | Alone | | | 22 | | REF | REF | REF |  |
|  | Together | | | 61 | | -19.35 | -57.97, 19.86 | .335 |  |
| **FTND** | | | |  | |  |  |  |  |
|  |  | | | 83 | | -6.33 | -13.70, 0.94 | .097 |  |
| **EQ5D** | | | |  | |  |  |  |  |
|  |  | | | 83 | | 33.92 | -65.99, 133.90 | .512 |  |
| **BSI-18** | | | |  | |  |  |  |  |
|  |  | | | 83 | | 15.42 | -12.77, 43.34 | .288 |  |
| **AUDIT** | | | |  | |  |  |  |  |
|  |  | | | 83 | | 3.09 | 0.17, 6.09 | .046 |  |
| **Diagnosis lung cancer** | | | |  | |  |  |  |  |
|  | No | | | 69 | | REF | REF | REF |  |
|  | Yes | | | 14 | | -22.40 | -77.00, 33.00 | .430 |  |
| **Diagnosis breast cancer** | | | |  | |  |  |  |  |
|  | No | | | 41 | | REF | REF | REF |  |
|  | Yes | | | 42 | | 18.97 | -16.43, 53.89 | .296 |  |
| **Cancer sites** | | | |  | |  |  |  |  |
|  | 1 | | | 69 | | REF | REF | REF |  |
|  | 2 or 3 | | | 14 | | 51.52 | 7.69, 95.98 | .027 |  |
| **Number of logins** | | | |  | |  |  |  |  |
|  |  | | | 56 | | -0.11 | -0.50, 0.29 | .596 |  |
| **Number of diary entries** | | | |  | |  |  |  |  |
|  |  | | | 56 | | -0.01 | -0.31, 0.29 | .949 |  |
| **Number of exercises** | | | |  | |  |  |  |  |
|  |  | | | 56 | | 0.39 | -3.04, 3.81 | .824 |  |

^a^A Bonferroni correction was applied based on 14 tests resulting in an alpha of 0.004.
^b^REF: reference category.

Table 3. Sensitivity analysis of the moderation analysis of study condition on the relationship between participant characteristics and smoking behavior.

|  | |  | Participant characteristic * RCT condition 7-day tobacco use at 6-month follow-up (N = 165) | | |
| --- | --- | --- | --- | --- | --- |
| Interaction with Condition | | n | B | CI | *P*^a^ |
| **Age (years)** | |  | 1.08 | -0.93, 3.11 | .298 |
|  | |  |  |  |  |
| **Gender** | |  |  |  |  |
|  | Men | 29 | REF^b^ | REF | REF |
|  | Women | 136 | 29.18 | -27.61, 86,43 | .321 |
| **Higher education** | |  |  |  |  |
|  | No | 121 | REF | REF | REF |
|  | Yes | 44 | -12.94 | -59.95, 34.34 | .594 |
| **Living situation** | |  |  |  |  |
|  | Alone | 48 | REF | REF | REF |
|  | Together | 117 | -7.51 | -51.13, 36.44 | .739 |
| **FTND** | |  | -4.21 | -12.48, 3.99 | .321 |
|  | |  |  |  |  |
| **EQ5D** | |  | 59.58 | -46.42, 165.74 | .277 |
|  | |  |  |  |  |
| **BSI-18** | |  | 4.58 | -33.60, 42.54 | .815 |
|  | |  |  |  |  |
| **AUDIT** | |  | 4.34 | -0.13, 8.81 | .062 |
|  | |  |  |  |  |
| **Diagnosis lung cancer** | |  |  |  |  |
|  | No | 142 | REF | REF | REF |
|  | Yes | 23 | 35.72 | -23.60, 95.29 | .245 |
| **Diagnosis breast cancer** | |  |  |  |  |
|  | No | 90 | REF | REF | REF |
|  | Yes | 75 | 17.53 | -23.17, 58.52 | .405 |
| **Cancer sites** | |  |  |  |  |
|  | 1 | 137 | REF | REF | REF |
|  | 2 or 3 | 28 | 86.13 | 34.52, 138.27 | *.002* |

^a^A Bonferroni correction was applied based on 11 tests resulting in an alpha of 0.0045.
^b^REF: reference category.
